# Microecological mechanisms of mountainous forest cultivated ginseng growth vigor and saponin accumulation, and the characterization of bionic microbial fertilizer

**DOI:** 10.3389/fmicb.2025.1548481

**Published:** 2025-05-13

**Authors:** Fengyu Pang, Yugang Gao, Zhenqi Zhuang, Changju Li, Yan Zhao, Qun Liu

**Affiliations:** ^1^College of Chinese Medicinal Materials, Jilin Agricultural University, Changchun, China; ^2^Institute of Botany, Jiangsu Province and Chinese Academy of Sciences (Nanjing Botanical Garden Mem. Sun Yat-Sen), Nanjing, China

**Keywords:** ginseng, growth vigor, saponin accumulation, microecological mechanisms, bionic microbial fertilizer

## Abstract

**Introduction:**

A study on the soil microecological mechanisms influencing the growth vigor and saponin accumulation of mountainous forest cultivated ginseng (MFCG) under various forest types.

**Methods:**

Using MFCG from different forest types as experimental material, the correlation and functional analysis of MFCG growth vigor, ginseng saponin content, and soil nutrient elements in their rhizosphere were conducted to clarify the soil microecological mechanisms by which different forest types affect the growth vigor and saponin accumulation of understory ginseng. Based on these microecological mechanisms, a bionic microbial fertilizer was developed and characterized.

**Results:**

The agronomic traits and saponin content (Re, Rc, Rb2, and Rb3) of MFCG in the *Pinus sylvestris* var. mongholica Litv. (PSV) group were significantly higher than those in the *Quercus mongolica* Fisch. ex Ledeb. (QMF) and *Larix gmelinii* (Rupr.) Kuzen (LGK) groups (*p* < 0.05). The total content of these four monomeric saponins in the PSV group was 35.1 and 45.56% higher than that in the QMF and LGK groups, respectively. Significant differences (*p* < 0.05) were observed between the PSV group and the QMF and LGK groups in terms of the rhizosphere soil microbial diversity and physicochemical indicators such as nutrient elements. The agronomic traits and saponin content of MFCG were positively correlated with chemical indicators in the rhizosphere soil, including Cu, Ca, Mg, Zn, B, Fe, Mo, Mn, Organic matter (OM), Available phosphorus (AP), Available nitrogen (AN), and Available potassium (AK). Based on the microbial diversity and nutrient elements positively correlated with MFCG in the rhizosphere soil, a bionic microbial fertilizer formula was optimized.

**Discussion:**

The microecological mechanism behind the growth vigor and saponin accumulation of understory ginseng involves an increase in beneficial microorganisms and nutrient elements, along with a reduction in harmful microorganisms and detrimental elements. The bionic microbial fertilizer promoted MFCG growth and saponin accumulation while improving soil nutrient levels, bulk density, and water-holding capacity.

## 1 Introduction

Ginseng (*Panax ginseng*) is globally recognized as a valuable medicinal plant resource (Liu et al., 2020). However, understory ginseng, as a perennial plant, has recently faced the threat of endangerment, with high-quality MFCG being particularly scarce and in high demand (Zhang et al., 2020). Currently, there are two main cultivation methods for ginseng: MFCG and Garden ginseng (Tong et al., 2021). Garden ginseng is typically cultivated artificially in shaded conditions in mountainous areas or farmlands, while MFCG is grown in the natural environment of mountain forests with relatively minimal human intervention. Garden ginseng is usually harvested within 4 to 6 years, whereas MFCG requires more than 15 years of growth in the wild environment before it can be harvested (Qu et al., 2023). Ginseng has a long growth cycle and slow saponin accumulation, which significantly limits its yield. Ginsenosides are one of the key active components in ginseng, and their content is influenced by various environmental factors, particularly the growing region and soil conditions (Chen et al., 2022). An optimal growing environment is a key factor in producing high-quality MFCG. However, there is still a lack of systematic analysis regarding the impact of different forest types on ginsenoside accumulation and variation, as well as the correlation between soil microecology and ginsenoside content under different forest types. Therefore, studying the growth characteristics of MFCG under different forest conditions, and developing bionic microbial fertilizer for understory cultivation based on this microecological mechanism, is of significant theoretical and practical importance for improving the ecological cultivation benefits of MFCG.

The growth of understory ginseng and saponin accumulation are significantly influenced by key elements and microbial communities in the soil. Studies have shown that macronutrients such as N, P, K, as well as micronutrients like Fe, Mn, and Zn, play a crucial role in regulating the physiological metabolism of understory ginseng. Certain microorganisms, such as nitrogen-fixing bacteria, phosphorus-solubilizing bacteria, and *Bacillus mucilaginosus*, can enhance ginsenoside levels by regulating secondary metabolic pathways (Mu et al., 2024). Ecological systems vary under different forest types, leading to differences in the soil environments of each forest type (Gajendiran et al., 2024). Literature has reported that the abundance of phosphorus-solubilizing bacteria and actinobacteria is higher in coniferous forest soils, which can significantly increase rhizosphere P content and saponin accumulation, while broadleaf forests are more suitable for the growth of acidophilic bacteria (Mitra et al., 2022). Changes in forest types not only affect the microbial community structure but also significantly influence the abundance of microorganisms involved in saponin biosynthesis. Microorganisms in the soil environment play a crucial role in organic matter decomposition, nutrient cycling, and plant growth (Hu et al., 2024). Previous studies have shown that different environments influence the composition and diversity of microbial communities in the soil (Jansson and Hofmockel, 2020). Rhizosphere microorganisms in the soil microecosystem are influenced by plant exudates, soil type, and environmental conditions (Tong et al., 2024). These microorganisms promote plant nutrient absorption through mechanisms such as organic matter decomposition, pH regulation, and the release of signaling molecules (Singh et al., 2022). Studies have shown that specific microbial communities in the rhizosphere play a particularly crucial role in saponin biosynthesis (Li K. et al., 2024). The rhizosphere is the microecological environment in the soil that is closest to the plant roots and where interactions with soil microorganisms occur (Bai et al., 2022). As soil microorganisms in the rhizosphere play a decisive role in plant growth and health, an increasing number of researchers are exploring the correlation between rhizosphere soil microorganisms and plants (Duret et al., 2024). Plant–soil feedback refers to the interaction between microorganisms and plants (Aqeel et al., 2023). Generally, the impact of rhizosphere microorganisms on plants can be classified into positive and negative feedback (Aqeel et al., 2023). Positive feedback from rhizosphere microbial communities can promote plant growth and development, as well as enhance the plant’s ability to resist abiotic stress (Ali et al., 2024). Specifically, the increase in rhizosphere fungal diversity plays a significant role in promoting soil aggregate stability, which is facilitated by the enhancement of root traits (Wang et al., 2024b).

Currently, the mechanisms by which MFCG cultivation under different forest types affects rhizosphere soil chemical properties, nutrients, and microbial communities are not yet well understood. Negative feedback from rhizosphere microbial communities can inhibit plant growth and development. For example, when plants are continuously grown in the same soil, the increase in harmful microorganisms leads to a decline in crop yield, which is a typical case of plant-microbe negative feedback. Co-limitation of elements is a dominant condition in many biological systems, and this is also true in soil systems (Kaspari and Powers, 2016). Elements play a dominant role in soil systems, and different environments influence the content of these elements in the soil. Therefore, the application of composite microbial fertilizer significantly improves the rhizosphere soil microecological environment, including increasing the abundance and diversity of beneficial microorganisms, promoting the cycling of key nutrients in the soil, and enhancing plant nutrient absorption. However, there are currently few detailed reports on the application of cultivated microbial fertilizers for understory ginseng. The interaction between plants and soil microecology is crucial, and applying appropriate methods to regulate the soil microecology is of significant importance for ginseng growth (Park et al., 2024). The growing environment of ginseng has a significant impact on the synthesis and accumulation of ginsenosides (Fang et al., 2024). In this study, we used high-performance liquid chromatography (HPLC) to measure the content of nine ginsenosides and assess the agronomic traits of MFCG under three different forest types. Microbial differences in the rhizosphere soil of ginseng under different forest types were analyzed using Illumina MiSeq high-throughput sequencing technology. Simultaneously, the chemical properties of rhizosphere soil in MFCG under different forest types were compared using various techniques, including inductively coupled plasma mass spectrometry (ICP-MS), the alkali diffusion method, the molybdenum-antimony colorimetric method, flame photometry, and the potassium dichromate volumetric method. This study analyzed the effects of different forest types on ginsenoside content and agronomic traits of MFCG. Through correlation analysis between soil microecology and ginsenoside content under various forest types, the patterns of ginsenoside bioaccumulation and the influence of rhizosphere soil microecology across different forest types were explored. Furthermore, beneficial microorganisms, elements, and soil nutrient conditions that promote ginseng growth were identified through correlation analysis, followed by the formulation of a bionic microbial fertilizer. This screening process was more systematic and targeted. Finally, the impact of the bionic microbial fertilizer on MFCG growth rate, quality, and soil conditions was evaluated. This provides a theoretical basis for optimizing ecological cultivation conditions to improve ginsenoside quality and yield, contributing to the standardization of ecological cultivation and management practices.

## 2 Materials and methods

### 2.1 Sample source

To eliminate the potential influence of ginseng age on the experimental results, MFCG of the same age (15 years), which is considered representative, was selected. To minimize the impact of ecological factors other than forest type, different forest types within the same geographic location were chosen to ensure comparable environmental conditions. This study was conducted in September 2023 in a forest area in Tonghua County, Jilin Province (East Longitude: 125.753°E, North Latitude: 41.677°N, Elevation: 475 m), at the same site across three forest types: PSV, LGK, and QMF. All MFCG samples were identified by Professor Gao Yugang from the College of Chinese Medicinal Materials, Jilin Agricultural University. Samples were collected from 15-year-old healthy MFCG plants and their rhizosphere soils under the three different forest types (Hold the base of the MFCG stem and gently shake to remove the remaining soil attached). Control soil samples were collected from areas under the same forest types where ginseng had not been cultivated (at a depth of 20 cm), and were homogenized. The control soil (non-MFCG cultivation area) was located 2 m away from the ginseng planting area. For each forest type, 20 MFCG samples were collected, with a detailed sample list provided in Supplementary materials (Supplementary Table S1). Among these, samples S1–S12 and their rhizosphere soils were used for ginsenoside content and soil property analysis, while samples S1–S60 were used to measure MFCG agronomic traits.

### 2.2 The determination of agronomic traits of MFCG and ginsenoside content under different forest types

The MFCG samples were rinsed thoroughly with distilled water, and surface water was absorbed with filter paper. Agronomic traits, including plant height, stem thickness, leaf length, main root length, root thickness, and weight (fresh weight), were measured. The samples were then air-dried at 40°C, and the dry weight was recorded. Each individual MFCG plant was ground to a particle size of less than 0.18 mm, and the contents of nine saponins were determined using the HPLC method. The method for determining ginsenosides was based on previously published protocols from our laboratory (Li X. et al., 2024). The High-Performance Liquid Chromatography (HPLC) conditions are as follows: The HPLC system used was an LC-2010 A. The chromatographic column was a Shimadzu C18 column (150 mm × 4.6 mm, 5 μm). The column temperature was maintained at 35°C, and the detection wavelength was set to 203 nm. The mobile phase consisted of water (A) and acetonitrile (B). The gradient elution program was as follows: 0–24 min, 18% B, 82% A; 24–26 min, 22% B, 78% A; 26–30 min, 26% B, 74% A; 30–50 min, 32% B, 68% A; 50–55 min, 33.5% B, 66.5% A; 55–65 min, 38% B, 62% A. The ginsenoside content was quantitatively determined based on standard calibration curves. The concentration of each individual ginsenoside was calculated from its peak area using the corresponding standard curve, and the total ginsenoside content was obtained by summing the concentrations of all quantified ginsenosides.

### 2.3 Analysis of soil nutrients and other physicochemical properties

The soil samples collected in Section 2.1 were quickly stored in an icebox and transported back to the laboratory. Each soil sample was sieved through a 2 mm mesh, and a portion of the sample was stored at 4°C for analysis of soil nutrients and other physicochemical properties. To prevent sample degradation, the storage period at 4°C was limited to a maximum of 14 days before analysis. The content of AN in the soil was determined using the alkaline hydrolysis diffusion method (You et al., 2014). The content of AP was determined using the molybdenum-antimony colorimetric method (Xiong et al., 2015). The content of AK was determined using the flame photometry method (Zhao et al., 2023). OM was determined using the potassium dichromate titrimetric method (Garcia-Pausas and Paterson, 2011). The content of calcium, magnesium, sulfur, iron, zinc, manganese, copper, boron, molybdenum, and chlorine, as well as the heavy metal elements Pb, Cr, Cd, Hg, and As, were determined using inductively coupled plasma mass spectrometry (ICP-MS) (Bidar et al., 2020).

### 2.4 Genomic DNA preparation, PCR amplification, and Illumina MiSeq sequencing of MFCG rhizosphere soil across different forest types

The soil samples collected in Section 2.1 were quickly stored in an ice box and transported back to the laboratory, where they were stored at −20°C for microbial analysis. Total DNA was extracted from 0.5 g of each rhizosphere soil sample using the SPINasy Soil DNA Kit (MP Biomedicals, LLC, USA) according to the manufacturer’s protocol. DNA concentration and purity were assessed using a 1% agarose gel and NanoDrop 2000 (Thermo Fisher Scientific, Massachusetts, USA). Broad-spectrum primer pair 338F (5′-ACTCCTACGGGAGGCAGCA-3′) and 806R (5′-GGACTACHVGGGTWTCTAAT-3′) were used to amplify the 16S rRNA V3 + V4 region, while ITS1F (5′-CTTGGTCAGGAGTAA-3′) and ITS2 (5′-GCTGCGTTTCATCATGC-3′) primers were used to amplify the ITS region for fungal evaluation. The RNA operator ITS region was combined with adapter and barcode sequences for soil bacterial and fungal community analysis (López-Aladid et al., 2023). The volume of each PCR reaction was 20 μL, containing 4 μL of 5 × FastPfu buffer, 2 μL of 2.5 mM dNTPs, 0.8 μL of 5 μM primers, 0.4 μL of FastPfu polymerase, and 10 ng of template DNA. The amplification conditions were as follows: initial denaturation at 95°C for 5 min, followed by 25 cycles of denaturation at 95°C for 30 s, annealing at 55°C for 30 s, and extension at 72°C for 40 s, with a final extension at 72°C for 7 min. The amplification products were quantified using enzyme labeling, and then combined in a 1:1 ratio. DNA purification was performed using the Omega DNA Purification Kit (Omega Bio-Tek, USA). The library was assessed using the QSep400 system, and after quality control, sequencing was performed on the Illumina NovaSeq 6,000 platform. DNA library construction and sequencing were carried out by Beijing Biomarker Technologies Co., Ltd.

### 2.5 Bioinformatics analysis

Raw sequencing data were filtered using Trimmomatic (version 0.33) software. Primer sequences were identified and removed using cutadapt (version 1.9.1), resulting in Clean Reads free of primer sequences. High-quality sequences were obtained for subsequent analysis. Denoising, paired-end sequence merging, and removal of chimeric sequences were performed using the dada2 method in QIIME2 2020.6, resulting in the final effective data (Non-chimeric Reads). Species annotation in the samples was counted using Operational Taxonomic Unit (OTU) sequences.

Spearman correlation coefficients were used to analyze the relationships between soil microbes and MFCG saponin content, as well as between soil chemical properties and MFCG saponin content and agronomic traits. Principal coordinate analysis (PCA) of the top 10 components was performed using R statistical software (v3.6.0). Picrust2 was used to predict the bacterial community functions, while FUNGuild was employed to predict the fungal community functions (Douglas et al., 2020). The analyses and visualizations were primarily conducted on BMKcloud.^1^

### 2.6 Preparation of forest floor bionic microbial fertilizer

Based on Sections 2.2–2.5, the optimal forest type for ginseng growth and saponin accumulation under the forest floor micro-ecosystem of *Pinus massoniana* was selected. The species *Bacillus cereus* and *Bacillus subtilis* from the *Firmicutes* were chosen as microbial strains. *Bacillus subtilis* and *Bacillus cereus* were isolated using the plate dilution method (Wang et al., 2024a). Rhizospheric soil samples were serially diluted with sterile water and plated on PDA agar medium, followed by incubation at 37°C for 24–48 h. Colonies with typical morphological characteristics were selected and purified. Molecular identification was performed by sequencing the 16S rRNA gene, and the obtained sequences were compared against the NCBI database to determine the bacterial species. Additionally, based on the selected types and concentrations of nutrients, a forest floor ginseng bionic microbial fertilizer formulation was developed by combining rice husk, peanut cake, cottonseed cake, rapeseed cake, bone meal, and wheat bran substrates (Sun et al., 2022), as per ginseng cultivation practices. The detailed formulation is provided in Supplementary Table S10. The process for preparing the bionic microbial fertilizer is as follows: Primary Fermentation: Inoculate a 1% volume of bacterial liquid with an OD_600_ value of 0.6 from two strains into PDB medium and incubate at 28°C for 24 h, until the OD_600_ reaches 0.6 (Daud et al., 2019). Secondary Fermentation: Inoculate the primary fermentation liquid at a 1% ratio into sterilized rice husks. After mixing, ferment at 28°C for approximately 7 days (Lee et al., 2012). Tertiary Fermentation: After the secondary fermentation, inoculate the fermented rice husks at a 1% ratio into the mixed substrate. Add an appropriate amount of sterile water, mix thoroughly, seal, and ferment at 28°C for 7 days. The viability of the microorganisms is verified by the colony counting method (CFU method), ensuring the viable count reaches 10^6^–10^8^ CFU/g (Bhuyan et al., 2023).

### 2.7 Characterization of forest floor bionic microbial fertilizer

Conventional forest floor planting methods were used in a study conducted at a site in Shiling Town, Siping City (longitude: 124.497°E, latitude: 43.167°N, elevation: 448 m). High, medium, and low-dose microbial fertilizer groups, along with a blank control group (CK), were set up. Each treatment group had a ginseng bed with dimensions of 1.5 m × 10 m. Prior to planting, element fertilizers were evenly applied first, followed by the addition of bio-based microbial fertilizers. Finally, the soil was thoroughly mixed by tilling to ensure uniform distribution of the fertilizers. The high-dose group received 743.89 g/m^2^ of microbial fertilizer, the medium-dose group received 371.94 g/m^2^, and the low-dose group received 185.97 g/m^2^. Throughout the entire experimental period, no additional chemical fertilizers, pesticides, or microbial inoculants were applied. This field management approach was designed to eliminate the influence of external agricultural inputs and ensure that any observed effects on MFCG were solely attributable to the bio-based microbial fertilizer treatment. The CK group received no microbial fertilizer. Each group used the same variety (Erma Ya) of two-year-old forest floor ginseng seedlings. Each ginseng bed consisted of 40 rows, with 10 seedlings per row (weighing 25 ± 1 g), and 1 replicate was set for every 5 rows. MFCG was transplanted on April 22, 2024, and harvested on September 27, 2024. Three ginseng plants were harvested from each replicate. By analyzing the agronomic traits of MFCG, including fresh weight, stem height, root length, main root length, fibrous root length, main root diameter, stem diameter, petiole length, leaf length, and leaf width, as well as the dehydration rate, saponin content, disease index, and soil parameters such as field water-holding capacity, bulk density, available nitrogen, available phosphorus, available potassium, and organic matter, this study characterizes the effects of bionic microbial fertilizer on understory ginseng and determines its optimal application rate.


$$\text{Disease index}\;(\%)=[\begin{array}{l} \sum (\begin{array}{l} \text{Number of plants}\;\mathrm{at}\\ \text{each disease severity}\\ \text{level}\times \text{corresponding}\\ \text{level value} \end{array})÷\\ \left(\begin{array}{l} \text{Total number of}\\ \text{surveyed plants}\times \\ \text{highest level value} \end{array}\right) \end{array}]\times 100$$

### 2.8 Statistical analysis

The data were analyzed using one-way analysis of variance (ANOVA) in SPSS software (version 26.0; SPSS Inc., Chicago, Illinois, USA). All values are expressed as means ± standard error (SE). Statistical analysis and graphing were performed using GraphPad Prism (version 8.0). Duncan’s multiple range test was used for significance testing, with a significance level set at *p* < 0.05.

## 3 Results

### 3.1 Agronomic traits of MFCG under different forest types

**Figure 1:**
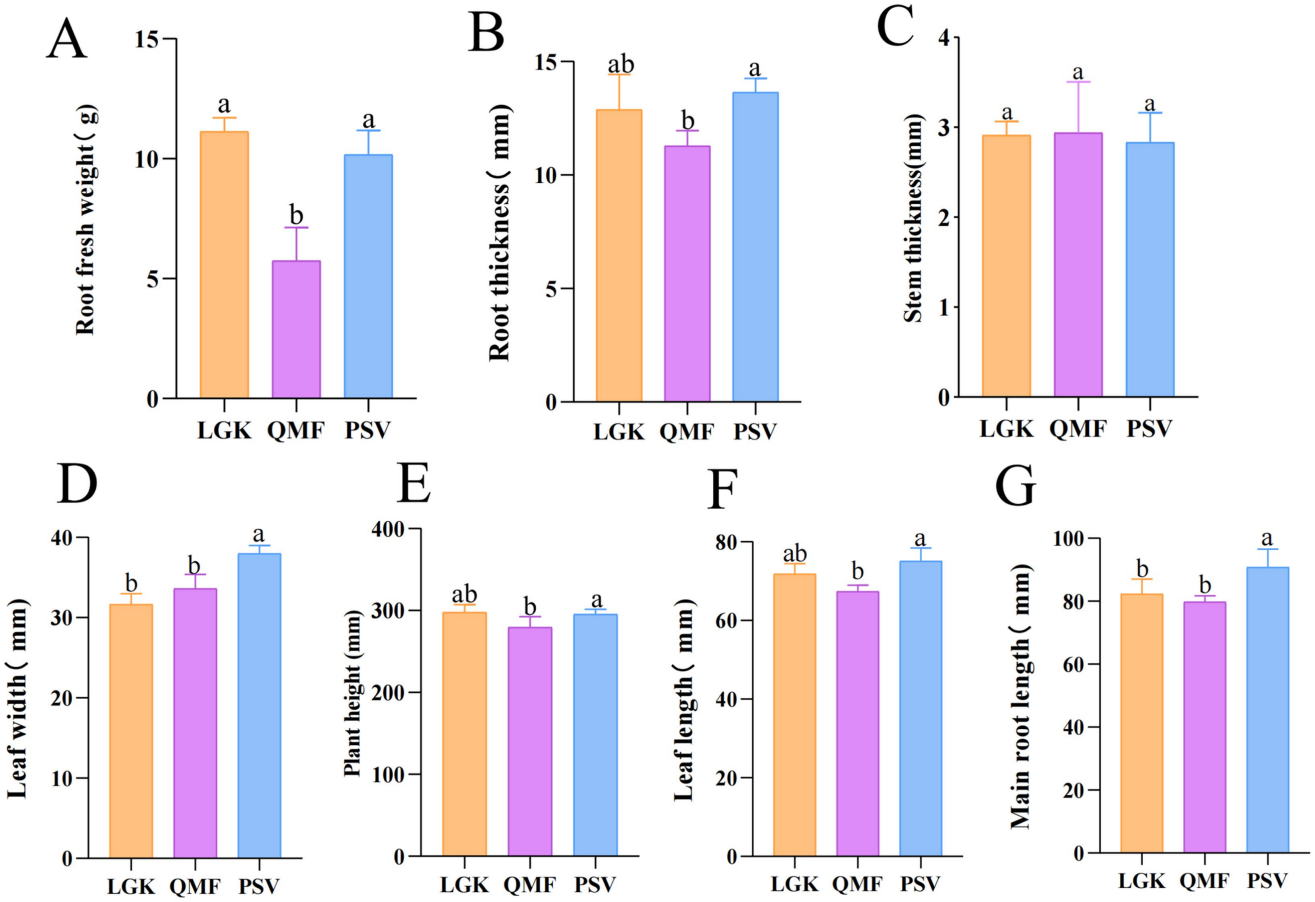
**(A–G)** Agronomic traits of MFCG. Different letters above the plots indicate significant differences between treatments (*n* = 20).

The agronomic traits of MFCG are shown in Supplementary Table S2. The root fresh weight (RFW), root thickness (RT), plant height (PH), and leaf length (LL) of MFCG in the PSV and LGK groups were significantly higher than those in the QMF group (*p* < 0.05) (Figures 1A,B,E,F). The leaf width (LW) and main root length (MRL) of MFCG in the PSV group were significantly higher than those in the LGK and QMF groups (*p* < 0.05) (Figures 1D,G). No significant differences were observed in stem thickness (ST) (*p* > 0.05) (Figure 1C). These results suggest that different forest types have a significant impact on certain agronomic traits of MFCG, with the *Pinus massoniana* forest type promoting MFCG growth.

### 3.2 Ginseng saponin content of MFCG under different forest types

**Figure 2:**
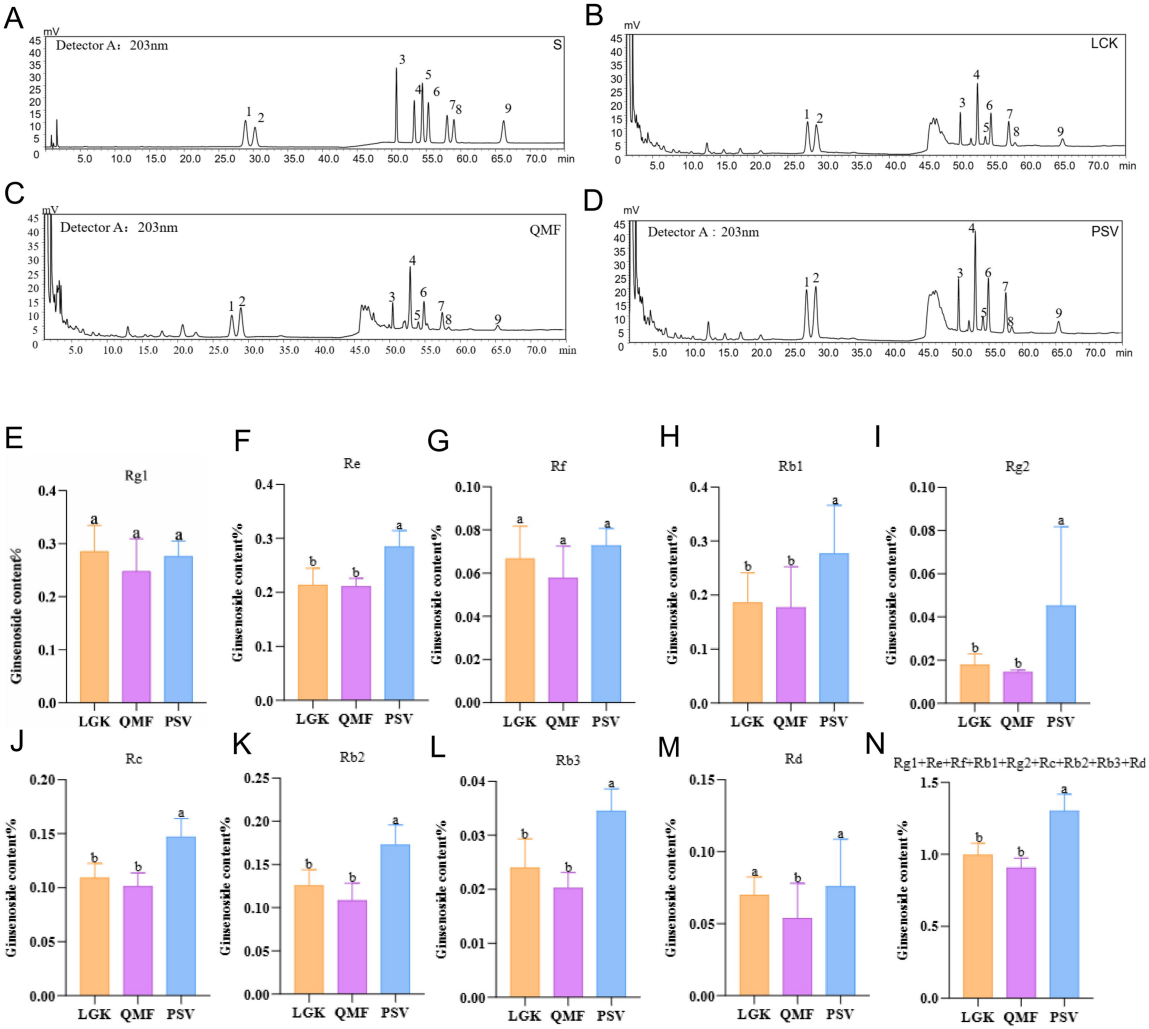
The concentrations of nine major ginsenosides in MFCG. **(A–D)** High-performance liquid chromatogram of ginsenosides detected from MFCG. S, Standard curves of nine ginsenosides; 1–9: Rg1, Re, Rf, Rb1, Rg2, Rc, Rb2, Rb3, Rd. detected from ginseng. **(E–N)** Ginsenoside content of MFCG. Different letters above the plots indicate significant differences between treatments (*n* = 4).

The HPLC chromatograms of MFCG ginseng saponins are shown in Figures 2A–D, and the saponin content is listed in Supplementary Table S3, with the specific saponin levels shown in Figures 2E–N. The content of individual ginseng saponins, including Re, Rb1, Rg2, Rc, Rb2, and Rb3, as well as the total content of 9 saponins, was significantly higher in the PSV forest type compared to the LGK and QMF types (*p* < 0.05) (Figures 2F,H–L). The content of the individual ginseng saponin Rd. was significantly higher in the PSV and LGK forest types than in the QMF type (*p* < 0.05) (Figure 2M). These results suggest that different forest types significantly influence certain agronomic traits of MFCG, with the *Pinus massoniana* forest type promoting its growth.

### 3.3 Statistical analysis of the OTUs in the MFCG rhizosphere soil microbial community

**Figure 3:**
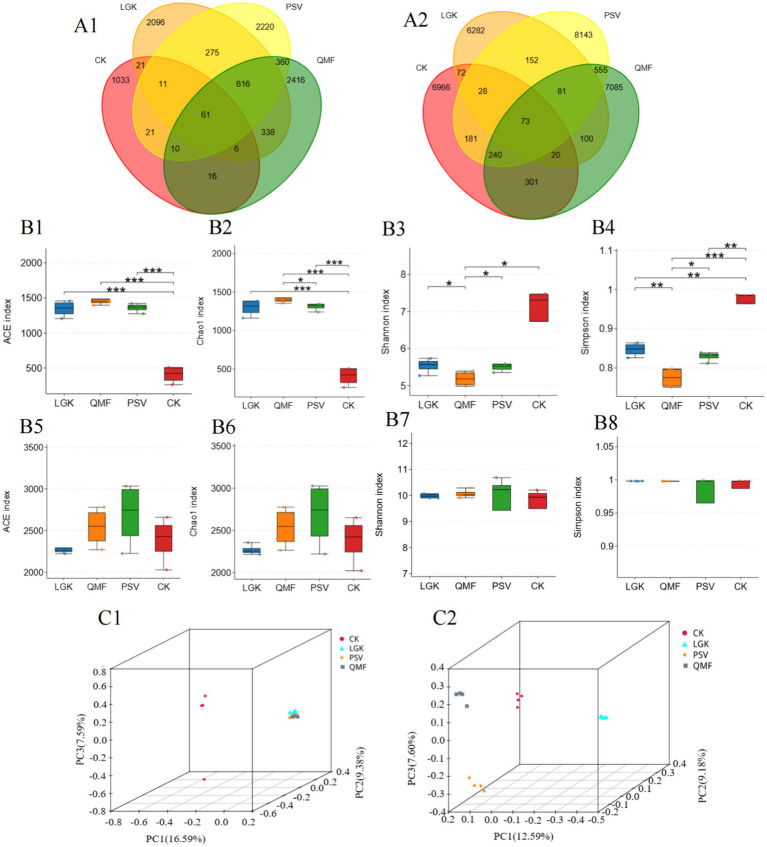
Effects of different forest types on the diversity of rhizosphere microbial communities in MFCG. **(A1,A2)** Venn diagrams of OTUs for soil fungi and bacteria, respectively. **(B1–B4)** ACE index, Chao 1 index, Shannon index, Simpson index for fungi. **(B5–B8)** ACE index, Chao 1 index, Shannon index, Simpson index for bacterias (*p < 0.05, **p < 0.01, *n* = 4). **(C1,C2)** PCoA results of fungal and bacterial communities, respectively (*p* < 0.05, *n* = 4).

Figure 3A shows that the number of shared fungal OTUs in the MFCG rhizosphere soil across the PSV, LGK, QMF, and CK groups was 61, accounting for 0.76% of the total OTUs. The number of unique OTUs in the LGK group was 2096, in the PSV group was 2,220, in the QMF group was 2,416, and in the control group was 1,033,indicating that the QMF group harbors the highest number of unique fungal OTUs. For bacterial OTUs, the number of shared OTUs in the MFCG rhizosphere soil across the PSV, LGK, QMF, and CK groups was 73, with the shared bacterial OTUs accounting for 0.26% of the total OTUs. The number of unique bacterial OTUs in the LGK group was 6,282, in the PSV group was 8,143, in the QMF group was 7,085, and in the control group was 6,966. These results suggest that the PSV group contains a higher number of unique bacterial species.

### 3.4 The impact of different forest types on the diversity of MFCG rhizosphere soil fungal and bacterial communities

The ITS rDNA sequence analysis of MFCG rhizosphere fungi under different forest types showed that the ACE index was significantly higher in all three forest types than in the CK group (*p* < 0.05), with the QMF group exhibiting the highest values among them (*p* < 0.05) (Figure 3B1). The Chao1 index followed a similar trend to the ACE index (Figure 3B2). The Shannon index was lower in all three forest types compared to the CK group, with the LGK group being significantly higher than the QMF and PSV groups (Figure 3B3), and the Simpson index showed a similar trend to the Shannon index (Figure 3B4). Based on 16S rRNA sequence analysis, it was found that the PSV group had significantly higher Ace, Chao1, Shannon, and Simpson indices than the QMF and LGK groups (Figures 3B5–B8) (*p* < 0.05). These results indicate that the bacterial richness and species diversity in the MFCG rhizosphere soil in the PSV forest type are higher than in the other two forest types. Overall, these results suggest that there are differences in the abundance and species diversity of MFCG rhizosphere fungi and bacteria across different forest types, with the QMF forest type having higher fungal richness, the LGK forest type having greater community diversity, and the PSV forest type having higher bacterial richness and greater community diversity.

### 3.5 The impact of different forest types on the structure of MFCG rhizosphere soil fungal and bacterial communities

Based on Bray-Curtis distances, PCoA analysis was performed on the fungal and bacterial communities in MFCG rhizosphere soil under different forest types. Due to the relatively low cumulative variance explained by PC1 and PC2, a 3D PCoA plot (Figures 3C1,C2) was used in this study to better visualize the differences in community structure among samples. Significant differences in microbial communities were observed between the groups (*p* < 0.05). The fungal community in the PSV group was significantly separated from those in the other groups (*p* < 0.05) (Figure 3C1). The bacterial community in the PSV group was significantly separated from the other groups, with significant separation also observed between the PSV and LGK groups, as well as between the PSV and CK groups (Figure 3C2). These results indicate that there are significant differences in the microbial community structure of MFCG rhizosphere soil under different forest types.

### 3.6 The community composition and dominant microbial taxa of MFCG rhizosphere soil under different forest types

**Figure 4:**
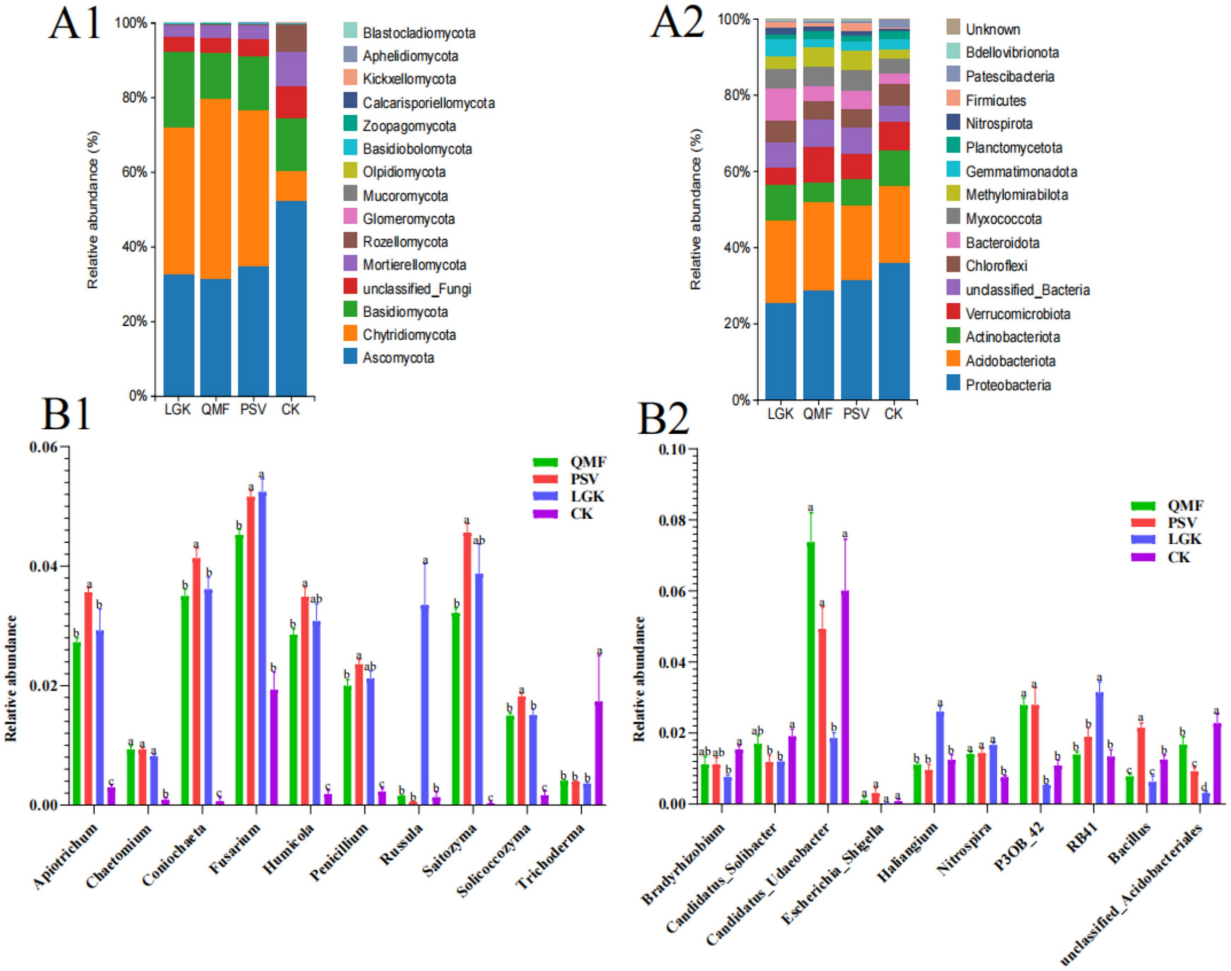
Analysis of soil microbial community composition and dominant communities among different forest types MFCG rhizosphere soil under different. **(Α1,A2)** Cumulative histograms of fungi and bacterial community composition (relative abundance (%) of ASVs at the phylum level). **(B1,B2)** Intergroup ANOVA of the top ten fungi and top ten bacteria with highest relative abundance at the genus level, respectively. Different letters above the plots indicate significant differences between treatments (*p* < 0.05, *n* = 4).

Microbial classification was performed based on amplicon sequence variants (ASVs). The relative abundance of the top 15 fungal communities at the phylum level in MFCG ginseng rhizosphere soil under different forest types is shown in Figure 4A1. Among them, *Ascomycota* and *Chytridiomycota* are the dominant fungal phyla in MFCG rhizosphere soil, with relative abundances ranging from 52% to 80%. The abundance of *Mucoromycota* in the QMF and PSV groups was 0.097 and 0.096%, respectively, which was significantly higher than that in the LGK and CK groups (*p* < 0.05). At the phylum level, the relative abundance of the top 15 bacterial communities in MFCG soil across different forest types is shown in Figure 4A2. *Proteobacteria*, *Actinobacteriota*, and *Acidobacteriota* are the dominant bacterial phyla in MFCG rhizosphere soil, with relative abundances ranging from 5.12% to 30.80%. The abundance of *Verrucomicrobiota* in the QMF group (9.16%) was significantly higher than in the CK group and the other two forest types (*p* < 0.05). The abundance of *unclassified_Bacteria* was significantly higher in all three forest types compared to the control group. The relative abundance of *Firmicutes* in the PSV group was significantly higher than in the other two forest types and the CK group (*p* < 0.05). We selected the top 10 fungal and bacterial genera with the highest relative abundance in the samples and performed an analysis of variance across the four treatment groups (Supplementary Tables S4, S5). The top 10 most abundant fungal genera were *Apiotrichum*, *Chaetomium*, *Coniochaeta*, *Fusarium*, *Humicola*, *Penicillium*, *Russula*, *Saitozyma*, *Solicoccozyma*, and *Trichoderma* (Figure 4B1). In the PSV forest type, the abundance of *Apiotrichum*, *Coniochaeta*, *Humicola*, *Penicillium*, *Saitozyma*, and *Solicoccozyma* was significantly higher than in the other two forest types and the CK group (*p* < 0.05). The top 10 most abundant bacterial genera were *Bradyrhizobium*, *Candidatus_Solibacter*, *Candidatus_Udaeobacter*, *Escherichia_Shigella*, *Haliangium*, *Nitrospira*, *P3OB_42*, *RB41*, *Bacillus*, and *unclassified_Acidobacteriales* (Figure 4B2). In the PSV forest type, the abundance of *Haliangium*, *RB41*, and *Bacillus* was significantly higher than in the other forest types and the CK group (*p* < 0.05).

### 3.7 The microbial community functions of MFCG rhizosphere soil under different forest types

**Figure 5:**
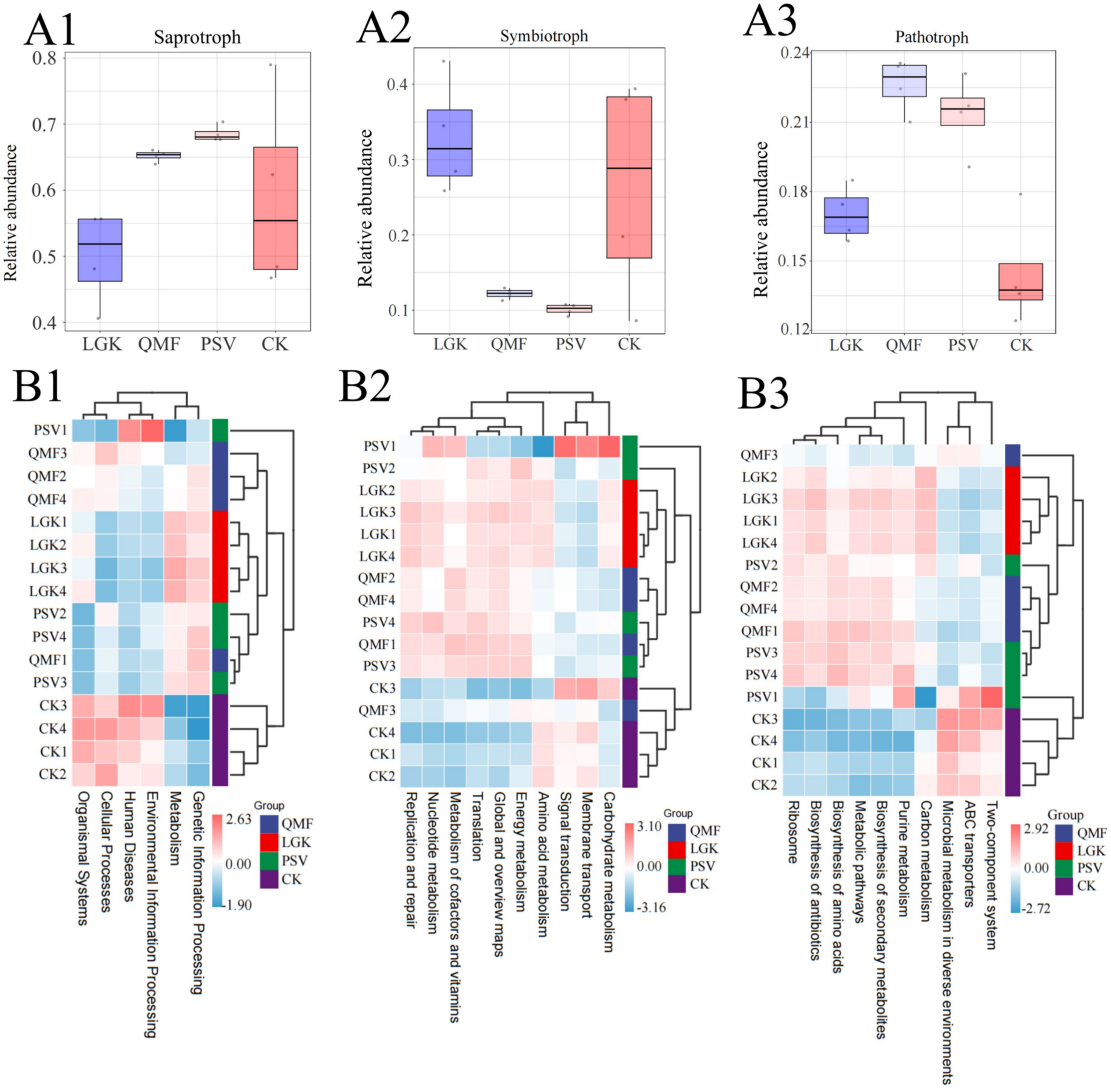
Functional predictions of fungi and bacteria in the rhizosphere soil of ginseng under different forest types. **(A1–A3)** Functions of fungal communities predicted by FUNGuild. **(B1)** Level 1 Functional prediction of bacterial communities. **(B2)** Level 2 Functional prediction of bacterial communities. **(B3)** Level 3 Functional prediction of bacterial communities (*n* = 4).

Using FUNGuild software, we predicted the nutritional and functional guilds of the fungal communities under different forest types (Supplementary Table S6). The microbial community functions varied across forest types. Based on nutritional modes, fungi were classified into three main guilds: saprotrophs, symbiotrophs, and pathotrophs (Figures 5A1–A3). In the PSV group, saprotrophs had the highest relative abundance. The relative abundance of symbiotrophs in the PSV and QMF groups was significantly lower than in the LGK and CK groups. The QMF group had the highest relative abundance of pathotrophs.

Using Picrust2, we predicted the 16S rRNA gene sequences (Supplementary Tables S7-1, S7-2, S7-3). The results show a comparison of KEGG pathway abundance between the three forest types and the control group (Figures 5B1–B3). In the first-level functional classification, the PSV forest type exhibited significant advantages in metabolism and genetic information processing, reflecting a stronger nutrient cycling and energy conversion capacity of the microbial community under the PSV forest type. The second-level functional classification further refined metabolic functions and environmental adaptation-related functions. Under the PSV forest type, carbohydrate metabolism, amino acid metabolism, and signal transduction functions were more prominent. The third-level functional analysis revealed that the PSV forest type had relatively higher abundances in metabolic pathways, purine metabolism, two-component systems, and ABC transporters. Among these, the abundance of two-component systems in the PSV group was approximately 10% higher than in the QMF and LGK groups, indicating that the microbial ecosystem in the PSV forest type is better adapted to environmental changes.

### 3.8 Correlation analysis of soil microbes in the rhizosphere of different forest types with MFCG ginsenoside content and agronomic traits

**Figure 6:**
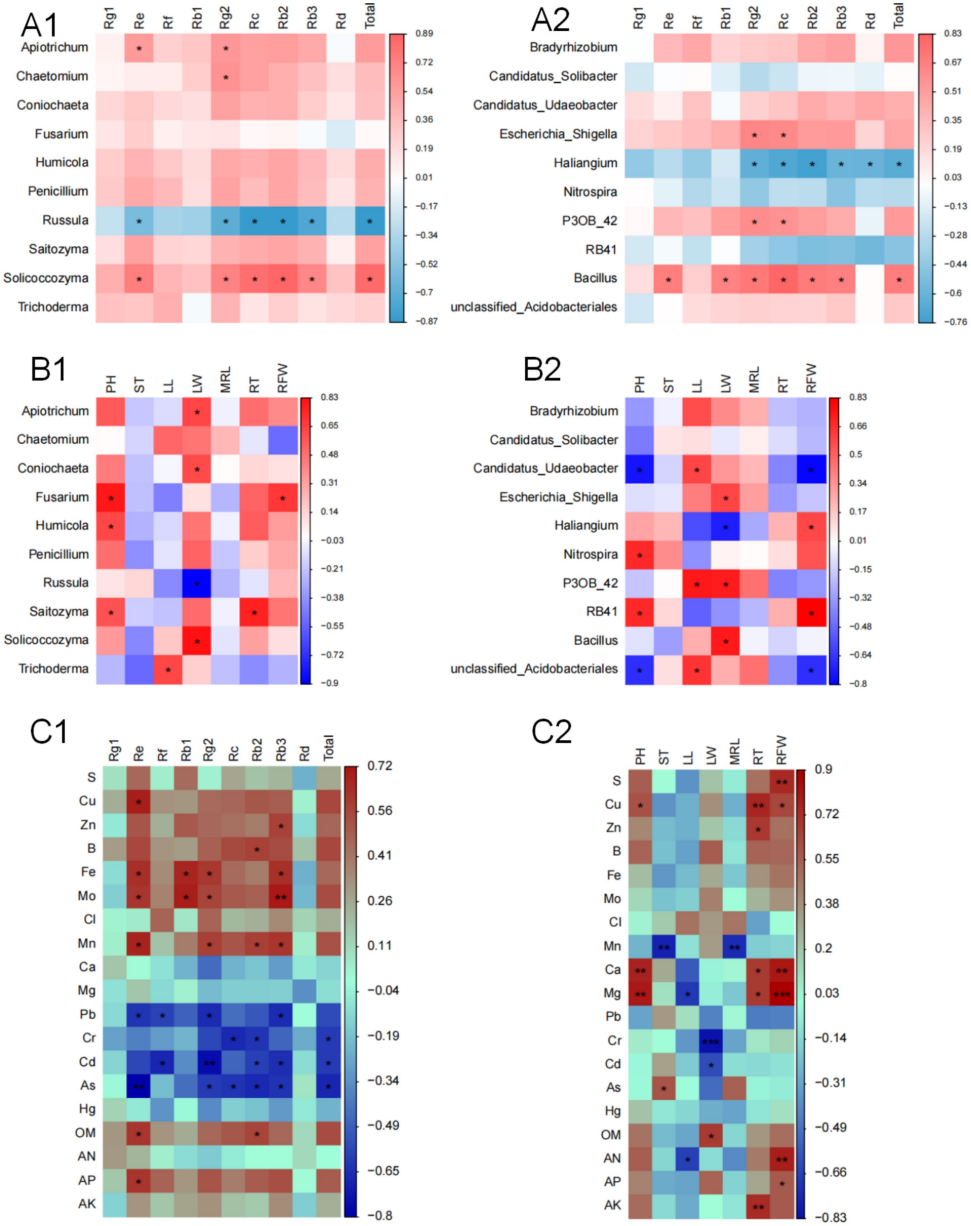
Correlation between Ginsenoside content, agronomic traits and rhizosphere microbial community based on Spearman’s correlation coefficient. **(A1,A2)** Analysis of the relationship between the top 10 fungi and bacteria and ginsenosides. **(B1,B2)** Analysis of the relationship between the top 10 fungi and bacteria and ginseng agronomic traits. RFW, Root fresh weight; RT, Root thickness; ST, Stem thickness; LW, Leaf width; PH, Plant height; LL, Leaf length; MRL, Main root length. **(C1,C2)** Correlation analysis of the chemical properties of MFCG rhizosphere soil with ginsenoside content and agronomic traits, respectively. OM, organic matter; AN, available nitrogen; AP, available phosphorus; AK, available potassium; Asterisks denote significant differences (*p < 0.05, **p < 0.01, ***p < 0.001, *n* = 4).

A correlation analysis was performed between the top 10 fungal and bacterial genera with the highest relative abundance and the MFCG ginsenoside content (Figures 6A1,A2). The sum of the nine major ginsenosides showed a significant positive correlation with the genus *Solicoccozyma*. Ginsenoside Rg2 content was significantly positively correlated with the genera *Apiotrichum*, *Chaetomium*, and *Solicoccozyma*. Ginsenoside Re content was positively correlated with *Apiotrichum* and *Solicoccozyma*. Ginsenosides Re, Rg2, Rc, Rb2, Rb3, and the sum of the nine ginsenosides were significantly negatively correlated with the fungal genus *Russula*. The sum of the nine major ginsenosides was significantly positively correlated with the bacterial genus *Bacillus* and significantly negatively correlated with *Haliangium*. Ginsenosides Rg2 and Rc content showed a significant positive correlation with the genera *Escherichia_Shigella*, *P30B_42*, and *Bacillus*. Ginsenosides Re, Rb1, Rb2, and Rb3 were all significantly positively correlated with the bacterial genus *Bacillus*.

Similarly, a correlation analysis was performed between the top 10 fungal and bacterial genera with the highest relative abundance and ginseng agronomic traits (Figures 6B1,B2). The PH of MFCG was significantly positively correlated with the fungal genera *Fusarium*, *Humicola*, and *Saitozyma*. The LW of MFCG was significantly positively correlated with *Apiotrichum*, *Coniochaeta*, and *Solicoccozyma*, and significantly negatively correlated with *Russula*. RT was significantly positively correlated with *Saitozyma*. RFW was significantly positively correlated with *Fusarium*. Regarding bacterial abundance, PH was significantly positively correlated with the genera *RB41* and *Nitrospira*, and significantly negatively correlated with *Candidatus_Udaeobacter*. LL was significantly positively correlated with *Candidatus_Udaeobacter* and *P30B_42*. LW was significantly positively correlated with *Escherichia_Shigella*, *P30B_42*, and *Bacillus*, and significantly negatively correlated with *Haliangium*. RFW was significantly positively correlated with *Haliangium* and *RB41*, and significantly negatively correlated with *Candidatus_Udaeobacter*.

### 3.9 Correlation analysis of the physicochemical properties of MFCG rhizosphere soil under different forest types with ginsenoside content and agronomic traits

The chemical properties of MFCG rhizosphere soil under different forest types (Supplementary Tables S8, S9) influence the quality and yield of ginseng. A correlation analysis was conducted between MFCG ginsenosides, key agronomic traits, and soil chemical properties (Figures 6C1,C2). The correlation between soil chemical properties and MFCG ginsenoside content is shown in Figure 6C1. Ginsenoside Re is significantly positively correlated with Cu, Fe, Mo, Mn, OM, and AP, and significantly negatively correlated with Pb and As. Rg2 and Rb3 are significantly positively correlated with Fe, Mo, and Mn, and significantly negatively correlated with Pb, Cd, and As. Rb2 is also significantly positively correlated with OM, and significantly negatively correlated with Cr, Cd, and As. Figure 6C2 shows the correlation between soil chemical properties and MFCG agronomic traits. RFW is significantly positively correlated with the contents of AN, AP, Mg, Ca, S, and Cu. RT is significantly positively correlated with Cu, AK, Zn, Ca, and Mg. MRL and ST are significantly negatively correlated with Mn. LW is significantly positively correlated with OM and significantly negatively correlated with Cr and Cd. pH is significantly positively correlated with Cu, Ca, and Mg.

### 3.10 The effect of bio-based microbial fertilizer on the agronomic traits of MFCG

The application of bio-based fertilizers significantly affected certain agronomic traits of MFCG (*p* < 0.05; Supplementary Figure 1). Bio-based fertilizers significantly increased the fresh weight of MFCG (*p* < 0.05). Compared to the control group, the fresh weight of the low, medium, and high dose bio-fertilizer groups increased by 20.08, 38.96, and 26.10%, respectively. The medium dose of bio-based fertilizer significantly increased the stem height of MFCG (*p* < 0.05), with a 21.43% increase in stem height compared to the control group, accelerating the growth of the aboveground portion of MFCG. Bio-based fertilizers also significantly increased the main root length of MFCG (*p* < 0.05), with the medium dose fertilizer group showing a 52.25% increase in main root length compared to the control group. The medium dose of bio-based fertilizer significantly increased the leaf length of MFCG (*p* < 0.05), with a 14.91% increase compared to the control group, indicating that the medium dose of bio-fertilizer had a notable promoting effect on the growth of the aboveground leaves.

### 3.11 The effect of bio-based microbial fertilizer on the disease incidence of MFCG

The application of bio-based microbial fertilizer significantly reduced the disease incidence of MFCG (*p* < 0.05; Supplementary Figure 1), with the medium-dose fertilizer group showing the most significant effect. Compared to the control group, the disease incidence in the medium-dose fertilizer group decreased by 57.89%, while the disease incidence in the low-dose and high-dose fertilizer groups decreased by 36.84 and 43.11%, respectively. In summary, the bio-based microbial fertilizer demonstrated effective disease suppression across all application rates, with the medium-dose treatment showing the most pronounced effect. These findings highlight the potential value of microbial fertilizers in promoting healthy cultivation of MFCG and provide a scientific foundation for optimizing dosage and application strategies. Future research should focus on the long-term regulatory mechanisms of microbial fertilizers on the soil micro-ecological environment, thereby supporting the development of high-yield, high-quality, and sustainable cultivation systems for MFCG.

### 3.12 Effects of bionic microbial fertilizer on MFCG saponin content, soil bulk density, field water-holding capacity, and nutrient levels

The application of three different doses of bio-based fertilizer significantly affected the ginsenoside content of MFCG (*p* < 0.05, Supplementary Figures 2, 3). The total content of nine ginsenosides ranked from highest to lowest as follows: medium dose > high dose > low dose > CK. Compared to MFCG seedlings before cultivation, the ginsenoside content of MFCG in the CK group, which was cultivated without bio-fertilizer, increased by 25.37% after 5 months of cultivation. Compared to the CK group, the total content of the nine ginsenosides in the low, medium, and high dose bio-fertilizer groups increased by 38.04, 96.2, and 38.4%, respectively.

The application of bio-based fertilizer significantly affected the soil bulk density and field water holding capacity (*p* < 0.05; Supplementary Figure 4). Compared to the CK group, the application of medium-dose bio-fertilizer significantly reduced the soil bulk density of MFCG cultivation. Regarding field water holding capacity, the effects of applying low, medium, and high doses of bio-fertilizer were all significant (*p* < 0.05) compared to the CK group, with the sequence of field water holding capacity in the soils planted with ginseng as follows: medium dose > low dose > high dose. The application of bionic microbial fertilizer had a significant impact on soil nutrient levels (*p* < 0.05; Supplementary Figure 4). Compared to the CK group, all three fertilizer application rates significantly increased soil organic matter, available nitrogen, and available phosphorus. The ranking of organic matter, available nitrogen, and available phosphorus content in the ginseng planting soil followed the order: high dose > medium dose > low dose > CK, while the ranking of available potassium content was medium dose > high dose > low dose > CK. This indicates that the application of bionic microbial fertilizer can improve soil structure and nutrient conditions, increase soil porosity and water retention capacity, and effectively promote MFCG growth.

## 4 Discussion

*Panax ginseng* C.A. Mey is primarily cultivated in two forms: MFCG and farmland-cultivated ginseng. Due to the implementation of China’s Natural Forest Protection Program, the availability of ginseng cultivated through deforestation has sharply declined, while the cultivation techniques for farmland ginseng have not yet fully matured. Consequently, the shortage of MFCG resources has intensified the imbalance between supply and demand, making the development of understory ginseng cultivation a widely accepted solution. Therefore, it is essential to investigate the relationships among the agronomic traits, ginsenoside content, and ecological factors associated with different forest types to identify the dominant ecological drivers influencing MFCG growth and ginsenoside accumulation. Elucidating the microecological mechanisms by which different forest types affect MFCG growth performance and ginsenoside biosynthesis will help guide the selection of suitable forest environments for MFCG ecological cultivation. This research also has implications for optimizing reforestation strategies by identifying forest types conducive to subsequent MFCG planting. Moreover, these findings provide a theoretical foundation for the development and characterization of bionic microbial fertilizers aimed at improving unsuitable forestlands for MFCG cultivation, ultimately promoting the advancement of ecological cultivation techniques for MFCG.

### 4.1 Forest type influenced the agronomic traits and ginsenoside content of MFCG

Agronomic traits (e.g., root length, plant height, and stem diameter) are critical indicators for assessing the growth status and yield potential of MFCG. The results of this study demonstrated significant differences in agronomic traits of MFCG under different forest types. Compared with the other two forest types, the PSV forest type resulted in significantly greater plant height, as well as increased root weight and root diameter in the underground portion of the plant. In addition, leaf width was relatively larger under the PSV forest type. Since leaves are essential sites for photosynthesis, their development is closely linked to the accumulation of active compounds in the roots, thereby affecting the quality and economic value of MFCG. This finding is consistent with previous research indicating that ecological factors significantly influence plant growth and development (Pagnussat and Gomez-Casati, 2024). It also provides important insights for addressing challenges such as the slow growth rate and long production cycle of MFCG.

Ginsenosides are key indicators for evaluating the quality of ginseng. In this study, MFCG cultivated under different forest types exhibited significant differences in ginsenoside content. Notably, MFCG grown under the PSV forest type showed significantly higher levels of individual ginsenosides—Re, Rb1, Rg2, Rc, Rb2, and Rb3—as well as the total sum of nine major ginsenosides, compared to those grown under LGK and QMF forest types. These results are consistent with the findings of Yang et al. (2018), who highlighted the influence of environmental factors on the accumulation of plant secondary metabolites. The pronounced impact of forest type on MFCG quality may be attributed to the unique soil microecology and specific microclimatic conditions of the PSV forest type, which are more conducive to the biosynthesis and accumulation of ginsenosides. This discovery provides valuable insights into addressing the challenges of slow ginsenoside accumulation and low ginsenoside content in young understory-cultivated ginseng.

### 4.2 Differences in soil microorganisms of MFCG under different forest types

Soil microorganisms, as an important component of the soil ecosystem, have a significant impact on the growth and quality of MFCG. We chose to present the group averages rather than the relative abundance of individual samples in Figures 4A1,A2, primarily to simplify the analysis and highlight the overall impact of different forest types on the MFCG rhizosphere microbial community. This approach helps reduce variation caused by individual differences, making group differences more apparent and ensuring that the results are more representative. Overall, there are significant differences in the abundance and species diversity of fungi and bacteria in the rhizosphere soil of MFCG under different forest types. Specifically, the bacterial richness is higher under the PSV forest type. This result is consistent with previous studies, such as those by Liao et al., who found that the soil microbial community structure varies across different vegetation types due to differences in litter quantity and nutrient composition (Liao et al., 2023). In addition, the microbial community structure in the ginseng rhizosphere showed significant differences under different forest types. This result may be related to the impact of MFCG rhizodeposits on microbial growth and reproduction (Yuan et al., 2022).

In terms of the microbial community composition in the MFCG rhizosphere soil, Ascomycota was the dominant fungal phylum in both the MFCG rhizosphere soil and the control group. This result is consistent with the data from Fang et al. (2022). Our study further confirmed that, compared to the CK group, the relative abundance of Ascomycota was significantly lower under the cultivated ginseng forest types. Ascomycota primarily acts as a saprophytic fungus in the soil, capable of decomposing various plant celluloses and hemicelluloses, thereby promoting the accumulation of soil organic matter and nutrient cycling (Egidi et al., 2019). Therefore, it is necessary to consider improving soil nutrient status by adding microbial fertilizers. In terms of bacterial communities, the abundance of Firmicutes in the PSV forest type (1.98%) was significantly higher than in the other two forest types and the CK group. This may be due to the higher soil nutrient levels in the PSV forest type compared to the other two forest types, a result consistent with the study by Song et al. (2021). Furthermore, at the bacterial genus level, the relative abundance of *Bacillus* in the PSV forest type was significantly higher than in the other two forest types and the CK group. *Bacillus* species such as *Bacillus subtilis* and *Bacillus licheniformis* are beneficial for ginseng growth. This may explain why MFCG agronomic traits and ginsenoside content were relatively higher in the PSV forest type compared to QMF and LGK. Investigating the differences in soil microbial communities under different forest types provides a foundation for the development of bionic microbial fertilizers for MFCG.

### 4.3 Differences in the soil chemical properties of MFCG under different forest types

Soil chemical properties are one of the key factors influencing plant growth (Lu et al., 2015). This study found that there are differences in soil chemical properties under different forest types. In terms of OM content, the soil OM and AN levels in the PSV forest type were significantly higher than those in the QMF and LGK groups. OM and AN are crucial for root development (Ma et al., 2024), which may be an important factor contributing to the better quality of MFCG grown under the PSV forest type. Trace elements play an important role in the growth and quality of ginseng (Wu et al., 2022). The levels of elements such as Ca, Fe, and Mg in the MFCG soil under the PSV forest type were significantly higher than those in the other two forest types. These elements can enhance root structure, improve nutrient absorption capacity, and increase stress resistance (Rolić et al., 2024). These combined factors may be the reason for the better growth of MFCG under the PSV forest type. The CK group data showed that the soil levels of AN, AP, AK, and OM were significantly higher than those in the MFCG rhizosphere soil, indicating that ginseng cultivation significantly depletes soil nutrients. This result is consistent with existing literature (Li C. et al., 2024; Zhan et al., 2024). Investigating the differences in soil chemical properties under different forest types provides a foundation for the development of bionic microbial fertilizers for MFCG.

### 4.4 Relationship between ginsenoside content, agronomic traits of MFCG, and rhizosphere microorganisms

The close interactions between plants and microbial communities have been well documented in numerous studies (Fitzpatrick et al., 2020). The correlation analysis between microbial communities and ginsenoside content in this study revealed that different fungal and bacterial genera play distinct roles in ginsenoside accumulation in MFCG. *Bacillus subtilis* and *Bacillus cereus*, identified through correlation screening, were confirmed to promote ginsenoside accumulation in MFCG. This is likely due to the dual functionality of beneficial microbes, which not only stimulate secondary metabolism of ginsenosides in a manner similar to pathogenic microorganisms, but also establish symbiotic relationships with ginseng, contribute to biological control, and enhance plant growth. Therefore, identifying such functional microorganisms is of particular importance.

The correlation analysis among major agronomic traits of MFCG revealed that the soil microbial community plays a significant role in either promoting or suppressing ginseng growth. The genus *Bacillus* was positively associated with leaf width of MFCG, which may enhance plant growth by improving the soil environment and increasing nutrient availability, thereby promoting root development and nutrient uptake (Kukla et al., 2022).

Therefore, manipulating the composition of soil microbial communities and optimizing microbe–plant interactions may serve as an effective strategy to enhance the quality and yield of MFCG. From a practical perspective, the correlation analysis between ginsenoside content, agronomic traits of MFCG, and rhizosphere microbial communities provides a scientific basis for the selection and development of efficient functional microbial fertilizers.

### 4.5 Characterization of the bio-mimetic microbial fertilizer

This study found that the PSV forest type is more favorable for the growth of undergrowth ginseng and the accumulation of saponins compared to the other two forest types. Based on the microecological mechanisms revealed by the effects of different forest types (QMF, LGK, PSV) on the growth rate and saponin accumulation of undergrowth ginseng, a formulation was developed to optimize the microecology of the undergrowth ginseng rhizosphere soil. This led to the creation of a bio-inspired microbial fertilizer that precisely supplements beneficial microorganisms (*Bacillus subtilis* and *Bacillus cereus*), essential nutrients (N, P, K, Mg, B, Fe, Cu, Zn, Mn, etc.), and improves soil physicochemical properties (organic matter, biocarbon, etc.). This represents a significant innovation in the research of microbial fertilizers for undergrowth ginseng. The MFCG bio-inspired microbial fertilizer has shown excellent effects in promoting ginseng saponin accumulation and the growth of agronomic traits. This could be attributed to the improvement of beneficial microorganisms, key elements, and soil nutrients in the MFCG planting soil, which enhances the microecological environment.

The ecological planting of MFCG is a fascinating area of research, especially with regard to the role mechanisms of litter, canopy closure, trees, temperature, and root exudates in the formation of the rhizosphere microecology in undergrowth ginseng soil under different forest types. There is vast potential for further exploration in this field. The applicability of bio-inspired microbial fertilizers in different regions and forest types, as well as the impact of heavy metals on MFCG saponin metabolism, are both exciting areas of research.

## 5 Conclusion

This study revealed the microecological mechanisms by which the three forest types—QMF, LGK, and PSV—influence the growth and ginsenoside accumulation of MFCG. Based on the characteristics of the optimal rhizosphere microenvironment, a bio-based microbial fertilizer was developed to precisely supplement beneficial microorganisms and essential nutrients, while improving soil physicochemical properties. Field trials demonstrated that the application of this fertilizer effectively enhanced MFCG growth and ginsenoside accumulation.

## Data Availability

The datasets presented in this study can be found in online repositories. The names of the repository/repositories and accession number(s) can be found in the article/Supplementary material.

## Footnotes

^1^ www.biocloud.net
